# Poor Sleep Quality and Its Influencing Factors Among Iranian Patients with Esophageal and Gastric Cancer

**DOI:** 10.34172/mejdd.2024.367

**Published:** 2024-01-31

**Authors:** Negin Maroufi, Masoudreza Sohrabi, Shima Mehrabadi, Farhad Zamani, Hossein Ajdarkosh, Sare Hatamian, Atefeh Bahavar, Parvin Hassanzadeh, Fahimeh Safarnezhad Tameshkel, Ali Gholami

**Affiliations:** ^1^Epidemiology & Biostatistics Department, School of Public Health, Neyshabur University of Medical, Sciences, Neyshabur, Iran; ^2^Gastrointestinal and Liver Diseases Research Center, Iran University of Medical Sciences, Tehran, Iran; ^3^Student Research Committee, Neyshabur University of Medical Sciences, Neyshabur, Iran; ^4^Department of Epidemiology, School of Public Health, Iran University of Medical Sciences, Tehran, Iran; ^5^Noncommunicable Diseases Research Center, Neyshabur University of Medical Sciences, Neyshabur, Iran

**Keywords:** Sleep, Pittsburg sleep quality index, Cancer, Esophageal, Gastric

## Abstract

**Background:**

Sleep quality is a notable factor of well-being. It also may play a role in the development and progression of chronic diseases and cancers. Therefore, this study was performed to investigate poor sleep quality and its influencing factors among Iranian patients with esophageal and gastric cancer.

**Methods:**

In this cross-sectional study, a total of 312 Iranian adult patients who suffered from esophageal and gastric cancers were employed from a gastrointestinal cancer-based cohort study conducted in a referral hospital in Tehran between 2015 and 2018. Persian version of the Pittsburg Sleep Quality Index (PSQI) was used to measure poor sleep quality. Univariate and multiple logistic regression models were applied to determine the related factors to poor sleep quality.

**Results:**

Of the participants, 203 (65.06%) were men, and 75.96% had gastric cancer. The mean age was 63.13±12.10 years. The results demonstrated that more than 62% of the patients had poor sleep quality. 148 (62.44%) patients out of 237 patients with gastric cancer had poor-quality sleep. Also, 46 (64.38%) patients out of 237 patients with esophageal cancer had poor-quality sleep. Based on the results of multiple logistic regression models, marital status has a negative association with poor sleep quality (odds ratio [OR]=0.32, *P*=0.015). In addition, having chronic disease (OR=2.16; *P*=0.028) and wealth index (OR=3.11, *P*=0.013; OR=3.81, *P*=0.003; OR=3.29, *P*=0.009; OR=3.85, *P*=0.003 for rich, moderate, poor, and poorest subgroups, respectively) had a positive association with poor sleep quality.

**Conclusion:**

The findings showed that about two-thirds of the patients studied were poor sleepers. Also, it was observed that marital status, chronic disease, and wealth index were important factors associated with poor sleep quality.

## Introduction

 The importance of sleep to human health cannot be overstated.^1^ Poor sleep quality can negatively impact health and cause disorders such as insulin resistance, type 2 diabetes mellitus, cardiovascular disease, cancer, and mortality.^2,3^ In recent decades, the shift to an urban lifestyle has adversely affected sleep duration and quality.^4,5^

 It is estimated that patients with cancer are three times more likely to experience sleep disturbances than the general population.^6,7^ Sleep problems have serious physical and psychological consequences for patients with cancer, such as lowered quality of life, depression, impaired functional status, and early mortality.^8,9^ Predisposing factors for cancer-related sleep disturbances are multifactorial, including stress associated with cancer diagnosis and treatment, chemotherapy, use of corticosteroids, sedatives, and monoclonal antibodies.^10^ Potential biological mechanisms contributing to sleep problems in patients with cancer require further investigation.

 Among the major public threats worldwide, cancer is noticeable due to its high mortality in recent years. Based on World Health Organization (WHO) statistics and GLOBOCAN data, gastric and esophageal cancer are, respectively, the fifth and eighth most common cancers worldwide.^11,12^ In this context, sleep disorders are observed in patients with gastric and esophageal cancer.^13^ As one of the most burdensome symptoms among patients with cancer and survivors, sleep problems are consistently reported. The findings of a systematic review showed that the prevalence of sleep disturbances and/or sleep disorders in cancer was up to 95%.^14^ Sleep disturbance decreases after treatment, although cancer survivors report sleep problems more frequently than their non-cancerous controls.^15^

 Sleep disorders have been demonstrated in clinical practice as a contributing factor to poor healing, longer hospital stays, higher medical costs, and a greater chance of recurrence of cancer. They also decrease the quality of life and cause other complications.^16,17^ As such, it is crucial that sleep disorders are identified in patients who are at high risk, such as patients with cancer. According to recent studies performed in patients with cancer, predisposing factors of sleep disturbances would include factors such as age, sex, sleep history, smoking, lower education, pain, retirement, distress, depression, anxiety, comorbidity of other diseases, cancer metastasis, and so on.^18-24^ In this regard, knowing the risk factors of sleep impairment can help identify the most susceptible patients and deliver support. Therefore, this study aimed to evaluate poor sleep quality and its influencing factors among Iranian patients with esophageal and gastric cancer.

## Materials and Methods

###  Participants

 The data of this cross-sectional study were obtained from the Firoozgar Gastrointestinal Cancer Cohort Study (FGCCS) in Tehran, Iran. The FGCCS was conducted in patients with gastrointestinal cancers referred to Firoozgar hospital from 2015 to 2018. The inclusion criteria were age of more than 18 years, Iranian patients without a history of any previous cancers or actual chemotherapy, and consent to participate in the study. Of all included patients registered in FGCCS, 315 patients complained of gastric and esophageal cancers regardless of their stages and location who were included in this study.

 All the participants agreed to participate in the study by confirming informed consent according to the Declaration of Helsinki (Ethical code: IR.NUMS.REC.1398.050).

###  Instruments

 In the current study, the Pittsburg Sleep Quality Index (PSQI) was used to measure sleep quality.^25^ PSQI has been translated into different languages, and the Persian (Farsi) version was used in this study. Several validations have been done for different groups in Iran.^26-30^ However, the validity and reliability of PSQI have been assessed and confirmed among Iranian patients with cancer.^31^ It contains 19 questions that indicate seven components. Each component demonstrates different areas of sleeping, including subjective sleep quality, sleep latency, sleep duration, habitual sleep efficiency, sleep disturbances, use of sleeping medications, and daytime dysfunction.

 The first component demonstrates the subjective sleep quality, which scores based on the overall sleep quality rating over the past month. The second component which describes sleep latency, the summation of the time duration takes to fall asleep at night and the inability to fall asleep within 30 minutes, which is ranked as 0 (0), 1 (1-2), 2 (3-4), and 3 (5-6). Sleep duration is defined as the third component and it is based on the hours of actual sleep per night. To obtain the habitual sleep efficiency as the fourth component which is represented as a percentage, the number of hours slept divided by the number of hours spent in bed. Component 5 demonstrates the sleep disturbance, which is evaluated based on the summation of a series of questions about having trouble sleeping during the past month due to different reasons such as feeling too cold or warm, having bad dreams, or so forth, and the summation will be placed into four categories from 0 to 3. Component 6 is defined as the use of sleeping medication (0: not during the past month, 1: less than once a week, 2: once or twice a week, 3: three or more times a week). The last component illustrates daytime dysfunction, which is calculated based on the total summation of two items: keeping up the enthusiasm to get things done and having trouble staying awake while driving, eating, or so forth, and it is ranked as 0 (0), 1 (1-2), 2 (3-4), and 3 (5-6). Finally, PSQI scores are from 0 to 21, with 0, 1, 2, and 3 points allotted to each of the components. An overall score greater than 5 will be considered to have poor sleep quality.^25^

###  Variables

 In the current study, sleep quality was considered the dependent variable. Other variables, such as age in years (continuous), sex (female vs. male), marital status (single/widow/divorce vs. married), years of education ( < 12 vs. ≥ 12), cancer diagnosis (esophagus or gastric), stage (1 to 4), body mass index (BMI) ( < 25 vs. ≥ 25, objectively measured by the nurse), residency (urban vs. rural), comorbidity to other chronic diseases (no vs. yes), current smoking status (no vs. yes), current opioid use (no vs. yes; self-reported data), alcohol consumption (categorized as “no” = never been drinking alcohol and “yes” = have been drinking alcohol; self-reported data), ethnicity (Fars vs. other), Wealth Index (WI) (poorest, poor, moderate, rich, and richest), and physical activity level (level 1 to level 4) were measured by a checklist as independent variables. The WI was calculated based on assets items of patients’ families, including telephone, mobile phone, fridge, microwave, personal computer, washing machine, bathroom, kitchen, toilet, car, motorcycle, house, and the number of rooms per capita as well as the infrastructure of the house; with use of principal component analysis.^32-34^ In order to collect the data, the sleep quality questionnaire and checklist of studied variables were filled out by a trained interviewer.

###  Statistical Analysis

 In this study, data analysis was performed using STATA 14.0. Number, percentage, mean, and standard deviation (SD) were used to illustrate the descriptive analysis of the study population’s sleep quality according to the studied variables. The Chi-square test and multiple logistic regression model were used to determine whether there is an association between studied variables and PSQI at univariate and multiple (adjusted model) levels. Odds ratio (OR) with confidence interval (CI) was presented to show the power of association between different studied variables and sleep quality. A P value < 0.05 was considered to be statistically significant.

## Results

 The study included 315 eligible patients who were interviewed to complete the sleep quality questionnaire and checklist of studied variables. We excluded three subjects because their sleep quality questionnaires were incomplete. As a consequence, 312 persons (men = 203 women = 109) with a mean age of 63.13 ± 12.10 years were included in our analysis. As presented in Table 1, among the study population, 75.96% and 24.04% were detected with gastric and esophageal cancers, respectively. Among them, 194 (62.18%) had poor sleep quality. The mean ± SD of age among individuals with good and poor sleep quality were 64.17 ± 11.69, and 62.50 ± 12.33, respectively. Table 1 illustrates the prevalence of good and poor sleep quality according to different studied variables. It is indicated that of patients with poor sleep quality, 148 (76.29%) and 46 (23.71%) are suffering from esophageal and gastric cancer, respectively.

**Table 1:** Demographic and behavioral characteristics of the study population based on sleep quality

**Variables**		**Total (n=312)** **No. (%)**	**Good sleep quality (n=118)** **No. (%)**	**Poor sleep quality (n=194)** **No. (%)**
Age (y)	< 65	160 (51.28 )	58 (49.15)	102 (52.58)
	≥ 65	152 (48.72)	60 (50.85)	92 (47.42)
Gender	Male	203 (65.06)	85 (72.03)	118 (60.82)
	Female	109 (34.94)	33 (27.97)	76 (39.18)
Marital Status	Single/widow/divorce	53 (16.99)	13 (11.02)	40 (20.62)
	Married	259 (83.01)	105 (88.98)	154 (79.38)
Years of education	˂12	239 (76.60)	92 (77.97)	147 (75.77)
	≥ 12	73 (23.40)	26 (22.03)	47 (24.23)
Cancer type	Gastric	237 (75.96)	89 (75.42)	148 (76.29)
	Esophagus	75 (24.04)	29 (24.58)	46 (23.71)
Stage of cancer	1	32 (10.26)	12 (10.17)	20 (10.31)
	2	51 (16.35)	15 (12.71)	36 (18.56)
	3	119 (38.14)	51 (43.22)	68 (35.05)
	4	97 (31.09)	32 (27.12)	65 (33.50)
	Missing	13 (4.17)	8 (6.78)	5 (2.58)
BMI	˂25	192 (61.54)	78 (66.10)	114 (58.76)
	≥ 25	120 (38.46)	40 (33.90)	80 (41.24)
Family local residency	Urban	277 (88.78)	105 (88.98)	172 (88.66)
	Rural	35 (11.22)	13 (11.02)	22 (11.34)
Comorbidity to other chronic diseases	Yes	222 (71.15)	74 (62.71)	148 (76.29)
	No	90 (28.85)	44 (37.29)	46 (23.71)
Current smoking status	Yes	47 (15.06)	19 (16.10)	28 (14.43)
	No	265 (84.94)	99 (83.90)	166 (85.57)
Current opioid use	No	270 (86.54)	100 (84.75)	170 (87.63)
	Yes	42 (13.46)	18 (15.25)	24 (12.37)
Alcohol consumption	No	299 (95.83)	112 (94.92)	187 (96.39)
	Yes	13 (4.17)	6 (5.08)	7 (3.61)
Ethnicity	Fars	114 (36.54)	39 (33.05)	75 (38.66)
	Other	198 (63.46)	79 (66.95)	119 (61.34)
WI	Poorest	60 (19.23)	20 (16.95)	40 (20.62)
	Poor	59 (18.91)	22 (18.65)	37 (19.07)
	Moderate	54 (17.31)	18 (15.25)	36 (18.56)
	Rich	50 (16.03)	20 (16.95)	30 (15.46)
	Richest	53 (16.98)	32 (27.12)	21 (10.83)
	Missing	36 (11.54)	6 (5.08)	30 (15.46)
Physical activity level	Level 1	81 (25.96)	29 (24.58)	52 (26.80)
	Level 2	63 (20.19)	28 (23.73)	35 (18.04)
	Level 3	101 (32.37)	35 (29.66)	66 (34.02)
	Level 4	62 (19.88)	24 (20.34)	38 (19.59)
	missing	5 (1.60)	2 (1.69)	3 (1.55)

Abbreviations: BMI, Body mass index; WI, Wealth index.

**Figure 1:**
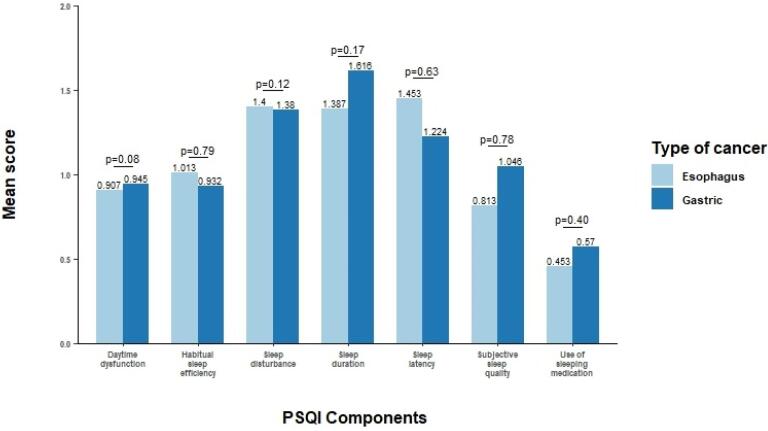
Mean score of each PSQI component among patients with esophageal and gastric cancers

Figure 1 presents the results of the independent sample *t* test between different sleep quality components and patients with cancer. Statistically, no significant result was observed between patients with esophageal and gastric cancers and different sleep quality components (*P* > 0.05). Table 2 indicates the results of univariate and multiple logistic regression models for patients with cancer and poor sleep quality. The results illustrate that age, years of education, stage of cancer, BMI, family local residency, current smoking status, current opioid use, alcohol consumption, and ethnicity do not have a statistically significant relation with poor sleep quality. A crude OR of 0.60 and 95%CI of 0.37-0.99, shows an association between sex and poor sleep quality, but it is not observed after adjustment for other variables. According to the adjusted regression model, individuals who lived alone had more than three times more chance to experience poorer sleep quality than those who were married. Also, patients with other chronic diseases were more likely to have poor sleep quality (OR: 2.16, 95%CI: 1.09-4.28).

**Table 2:** The association between studied factors and sleep quality in patients with esophageal and gastric cancers using univariate and multiple logistic regression models)

**Variables**	**Sleep Quality**	**Crude**			**Adjusted**		
		**OR**	**95% CI**	***P*** ** value**	**OR**	**95% CI**	***P*** ** value**
Age (years)	< 65	Reference	-	-	Reference	-	-
	≥ 65	0.87	0.55–1.38	0.557	0.92	0.52–1.63	0.782
Gender	Female	Reference	-	-	Reference	-	-
	Male	0.60	0.37–0.99	0.045	0.82	0.42–1.62	0.573
Marital Status	Single/widow/divorce	Reference	-	-	Reference	-	-
	Married	0.48	0.24–0.93	0.031	0.32	0.13–0.80	0.015
Years of education	˂12	Reference	-	-	Reference	-	-
	≥ 12	1.13	0.65–1.95	0.657	1.83	0.89–3.75	0.098
Stage of cancer	1	-	-	-	-	-	-
	2	1.44	0.56–3.67	0.445	1.75	0.58–5.28	0.320
	3	0.80	0.36–1.78	0.586	1.16	0.43–3.17	0.766
	4	1.22	0.53–2.80	0.641	1.24	0.46–3.35	0.674
BMI	˂25	Reference	-	-	Reference	-	-
	≥ 25	1.37	0.85–2.20	0.197	1.67	0.93–3.01	0.086
Family local residency	Rural	Reference	-	-	Reference	-	-
	Urban	0.97	0.47–2.00	0.930	0.81	0.35–1.90	0.636
Chronic disease	No	Reference	-	-	Reference	-	-
	Yes	1.91	1.16–3.15	0.011	2.16	1.09–4.28	0.028
Current smoking status	No	Reference	-	-	Reference	-	-
	Yes	0.88	0.47–1.65	0.690	1.07	0.45–2.56	0.877
Current opioid use	Negative	Reference	-	-	Reference	-	-
	Positive	0.78	0.40–1.52	0.470	0.93	0.42–2.07	0.868
Alcohol consumption	Negative	Reference	-	-	Reference	-	-
	Positive	0.70	0.23–2.13	0.529	0.44	0.12–1.57	0.208
Ethnicity	Fars	Reference	-	-	Reference	-	-
	Other	0.78	0.48–1.27	0.319	0.80	0.45–1.41	0.439
WI	Richest	Reference	-	-	Reference	-	-
	Rich	2.28	1.04–5.03	0.040	3.11	1.27–7.62	0.013
	Moderate	3.05	1.38–6.71	0.006	3.81	1.56–9.32	0.003
	Poor	2.56	1.19–5.49	0.016	3.29	1.35–8.03	0.009
	Poorest	3.05	1.41–6.57	0.004	3.85	1.57–9.44	0.003

Abbreviations: OR, odds ratio; CI, confidence interval; BMI, body mass index; WI, wealth index.

 Moreover, a significant association was observed between different levels of WI and poor sleep quality (OR = 3.85, 95%CI: 1.57-9.44 in poorest, OR = 3.29, 95%CI: 1.35-8.03 in poor, OR = 3.81, 95%CI: 1.56-9.32 in moderate, OR = 3.11, 95%CI: 1.27-7.62 in poor).

## Discussion

 In this study, the quality of sleep and factors affecting it in patients with esophageal and stomach cancers were investigated. We revealed that 62.18% of the study population had poor sleep quality. In addition, comorbidity to other chronic diseases, WI, and marital status had a significant relationship with sleep quality after adjusting for other studied variables. Also, there was no significant relationship between sex and sleep quality.

 Regarding the rate of poor sleep quality, our finding is almost consistent with other results. Former studies have demonstrated a poor sleep quality prevalence of 69.3%, 73.5%, 64%, and 50.8%.^35-38^ In a recent meta-analysis of data of 46,279 patients with different cancers, the rate of sleep disturbance reached to 60.7%.^39^ As the Petersburg scale indicated the sleep status for one month before the interview, environmental factors in our patients may be more weighted. One of the reasons that the prevalence of sleep disorders is different among studies relates to the conceptualization and operationalization of poor sleep that refers to undefined sleep disorders instead of a unique definition.^40^ In this regard, other factors such as socioeconomic and anxiety levels may influence patients before cancer diagnosis.

 According to our study, the mean scores of the components of sleep quality, including daytime dysfunction, habitual sleep efficiency, sleep disturbance, and use of sleeping medication, are approximately the same among patients with esophageal and gastric cancers (Figure 1). This lack of significant difference can be due to the presence of similar symptoms in both cancers, such as nausea, vomiting, and reflux.^41,42^ Moreover, it could be indicated that the type of upper gastrointestinal cancer is not an influencing factor in PSQI. However, this is not a fact in other cancers. The cause of this similarity is not clear but may be related to their pathogenesis, such as diet and other environmental related factors.

 Univariate analysis showed that sex was associated with poor sleep quality. By the way, when the data were adjusted, this association was not significant. Although there are a few studies evaluating sleep quality based on sex among patients with cancer, many of them have also found that women are more prone to sleep problems.^43-46^ Some factors, such as psychological issues, aging, hormonal status, and physical activities, may influence this outcome. The hormonal status may cause sleep quality problems for female patients. Higher estrogen levels may protect against sleep problems, according to a systematic review.^47^ At the same time, according to the meta-analysis conducted in 2021, controversial results were observed. This study reports that women sleep longer and more efficiently than men.^48^ One of the reasons for this discrepancy between the mentioned studies could be the different sample sizes in various studies. Also, the different number of variables for adjustment could be another reason for the observed discrepancy.

 In accordance with the other studies, our results showed that educational levels, BMI, physical activity level, family local residency, ethnicity, current smoking status, current opioid use, and alcohol consumption are not associated with sleep quality.^35,49,50^ There is no consensus regarding the association of age with sleep among patients with cancer; while some studies show an association with sleep distribution,^51-57^ the Ratcliff’s study did not find the same association.^49^

 As we revealed in the present study, the percentage of poor sleepers among alone individuals was higher than among married individuals, and in this regard, it was observed that marital status was associated with sleep quality. Contrary to our results, the findings of Endeshaw and colleagues’ study showed that the percentage of poor sleepers among married individuals was higher than among alone individuals and, in this regard, observed that marital status was associated with sleep quality.^56^ However the results of Ratcliff’s study showed that marital status was not associated with sleep quality in patients with breast cancer.^49^ According to the findings of our study and other mentioned studies, it seems that marital status is an effective factor in sleep quality in cancer patients, but more studies are needed in this field.

 Regarding the presence of chronic diseases, we revealed a significant relationship between sleep quality and comorbidity to other chronic diseases. The former studies by Hsu and colleagues and von Ruesten et al found a relationship between sleep duration and quality with chronic diseases.^58,59^ Chronic diseases always causes pain, contusion, and exhaustion, which will affect patients’ daily lives due to having trouble sleeping at night and making them sleepy during the daytime.

 Also, as shown in our study’s results, the prevalence of poor sleep quality in people with weak economic status (poorest and poor based on WI) was higher than in people with good economic status (richest and rich based on WI). It may be due to good life standards and easier access to hospitalization, medication, and cure. The association between economic status and sleep quality was indicated previously.^35,49^

 We did not find any association between the stage of cancer and poor sleep quality. Ratcliff and colleagues explored the association between sleep quality and the stage of cancer in a population with a specific type of cancer, and the results acted in accordance with the current study.^49^ Also, some other studies demonstrated that the stage and type of cancer were not relevant to sleep quality.^36,60^ Poor sleep quality is marked by a decreased number of sleep hours (sleep duration), a longer time it takes for you to fall asleep (sleep latency), nighttime awakenings (sleep disruptions), and the inability to function without naps throughout the day (daytime dysfunction).^61^ These symptoms are associated with insomnia, and they can occur alone or in combination, affecting sleep quality and daily functioning both at night and during the day.^62^ A sleep disorder may present with one or more of these symptoms, but they can be found in more than one sleep disorder at the same time.^63^

 This study faces some limitations that should be considered before interpretation of results. In the data-gathering phase, some questionnaires were fulfilled by the patients’ accompaniments due to the patients’ serious medical conditions and disabilities to respond. The present study come from of a cross-sectional point of view that has own limitation. However, this study had some strengths points; it is one of the few studies in this regard with substantial number of patients in our region. Also we considered the confounding factors such as comorbidities, richness status, family support or behavior factors. Finally, the results can be used as a hypothesis for future works to investigate the sleep quality as well as its influencing factors among general population at risk of gastrointestinal cancers or patients with cancer accurately.

## Conclusion

 Based on the results of the current study, it has been found that about two-thirds of patients with cancer suffer from poor sleep quality regardless of their cancer type and stage. It has been observed that there is a strong association between poor sleep quality and some influencing factors, including comorbidity to other chronic diseases, WI, and marital status. Therefore, it is essential to pay more attention to these influencing factors by providing more accessible and affordable access to hospitalization, remedies, and medications, reducing the symptoms and effects of prior chronic diseases, and having someone to live with them as a partner.

## References

[1] Billings ME, Hale L, Johnson DA (2020). Physical and social environment relationship with sleep health and disorders. Chest.

[2] Koo DL, Nam H, Thomas RJ, Yun CH (2018). Sleep disturbances as a risk factor for stroke. J Stroke.

[3] Besedovsky L, Lange T, Haack M (2019). The sleep-immune crosstalk in health and disease. Physiol Rev.

[4] Zomers ML, Hulsegge G, van Oostrom SH, Proper KI, Verschuren WMM, Picavet HSJ (2017). Characterizing adult sleep behavior over 20 years-the population-based Doetinchem Cohort Study. Sleep.

[5] Matricciani L, Bin YS, Lallukka T, Kronholm E, Dumuid D, Paquet C (2017). Past, present, and future: trends in sleep duration and implications for public health. Sleep Health.

[6] Chen YC, Lin CY, Strong C, Li CY, Wang JS, Ko WC (2017). Sleep disturbances at the time of a new diagnosis: a comparative study of human immunodeficiency virus patients, cancer patients, and general population controls. Sleep Med.

[7] Palesh OG, Roscoe JA, Mustian KM, Roth T, Savard J, Ancoli-Israel S (2010). Prevalence, demographics, and psychological associations of sleep disruption in patients with cancer: University of Rochester Cancer Center-Community Clinical Oncology Program. J Clin Oncol.

[8] Stone CR, Haig TR, Fiest KM, McNeil J, Brenner DR, Friedenreich CM (2019). The association between sleep duration and cancer-specific mortality: a systematic review and meta-analysis. Cancer Causes Control.

[9] Al Maqbali M, Hughes C, Rankin J, Dunwoody L, Hacker E, Gracey J (2021). Fatigue and sleep disturbance in Arabic cancer patients after completion of therapy: prevalence, correlates, and association with quality of life. Cancer Nurs.

[10] Grayson S, Sereika S, Harpel C, Diego E, Steiman JG, McAuliffe PF (2022). Factors associated with sleep disturbances in women undergoing treatment for early-stage breast cancer. Support Care Cancer.

[11] Mogavero MP, DelRosso LM, Fanfulla F, Bruni O, Ferri R (2021). Sleep disorders and cancer: state of the art and future perspectives. Sleep Med Rev.

[12] Rawla P, Barsouk A (2019). Epidemiology of gastric cancer: global trends, risk factors and prevention. Prz Gastroenterol.

[13] Lagergren P, Johar A, Rosenlund H, Arnberg L, Haglund L, Ness-Jensen E (2021). Severe reflux, sleep disturbances, and health-related quality of life after esophageal cancer surgery. J Cancer Surviv.

[14] Büttner-Teleagă A, Kim YT, Osel T, Richter K (2021). Sleep disorders in cancer-a systematic review. Int J Environ Res Public Health.

[15] Slade AN, Waters MR, Serrano NA (2020). Long-term sleep disturbance and prescription sleep aid use among cancer survivors in the United States. Support Care Cancer.

[16] Song C, Zhang R, Wang C, Fu R, Song W, Dou K (2021). Sleep quality and risk of cancer: findings from the English longitudinal study of aging. Sleep.

[17] Wennberg AMV, Wu MN, Rosenberg PB, Spira AP (2017). Sleep disturbance, cognitive decline, and dementia: a review. Semin Neurol.

[18] Lee K, Cho M, Miaskowski C, Dodd M (2004). Impaired sleep and rhythms in persons with cancer. Sleep Med Rev.

[19] Savard J, Morin CM (2001). Insomnia in the context of cancer: a review of a neglected problem. J Clin Oncol.

[20] Davidson JR, MacLean AW, Brundage MD, Schulze K (2002). Sleep disturbance in cancer patients. Soc Sci Med.

[21] Savard J, Simard S, Hervouet S, Ivers H, Lacombe L, Fradet Y (2005). Insomnia in men treated with radical prostatectomy for prostate cancer. Psychooncology.

[22] Coles T, Tan X, Bennett AV, Sanoff HK, Basch E, Jensen RE (2018). Sleep quality in individuals diagnosed with colorectal cancer: factors associated with sleep disturbance as patients transition off treatment. Psychooncology.

[23] da Silva Souza RC, Dos Santos MR, das Chagas Valota IA, Sousa CS, Costa Calache ALS (2020). Factors associated with sleep quality during chemotherapy: an integrative review. Nurs Open.

[24] Akman T, Yavuzsen T, Sevgen Z, Ellidokuz H, Yilmaz AU (2015). Evaluation of sleep disorders in cancer patients based on Pittsburgh Sleep Quality Index. Eur J Cancer Care (Engl).

[25] Buysse DJ, Reynolds CF, 3rd 3rd, Monk TH, Berman SR, Kupfer DJ (1989). The Pittsburgh Sleep Quality Index: a new instrument for psychiatric practice and research. Psychiatry Res.

[26] Farrahi Moghaddam J, Nakhaee N, Sheibani V, Garrusi B, Amirkafi A (2012). Reliability and validity of the Persian version of the Pittsburgh Sleep Quality Index (PSQI-P). Sleep Breath.

[27] Chehri A, Brand S, Goldaste N, Eskandari S, Brühl A, Sadeghi Bahmani D (2020). Psychometric properties of the Persian Pittsburgh Sleep Quality Index for adolescents. Int J Environ Res Public Health.

[28] Nazifi M, Mokarami H, Akbaritabar A, Kalte HO, Rahi A (2014). Psychometric properties of the Persian translation of Pittsburgh Sleep Quality Index. Health Scope.

[29] Mohammad Gholi Mezerji N, Naseri P, Omraninezhad Z, Shayan Z (2017). The reliability and validity of the Persian version of Pittsburgh Sleep Quality Index in Iranian people. Avicenna J Neuropsychophysiol.

[30] Farrahi J, Nakhaee N, Sheibani V, Garrusi B, Amirkafi A (2009). Psychometric properties of the Persian version of the Pittsburgh Sleep Quality Index addendum for PTSD (PSQI-A). Sleep Breath.

[31] Shahidi J, Khodabakhshi R, Yahyazadeh SH, Amini MG, Nosrati H (2007). Quality of sleep in cancer patients: evidence from Persian translation of Pittsburg Sleep Quality Index. Austral Asian J Cancer.

[32] Rutstein SO. The DHS Wealth Index: Approaches for Rural and Urban Areas. Calverton, Maryland: Macro International; 2008.

[33] Creation of a Wealth Index. World food Programme. 2017. Available from: https://docs.wfp.org/api/documents/WFP-0000022418download/.

[34] Asadi-Lari M, Moosavi Jahromi L, Montazeri A, Rezaee N, Haeri Mehrizi AA, Shams-Beyranvand M (2019). Socio-economic risk factors of household food insecurity and their population attributable risk: a population-based study. Med J Islam Repub Iran.

[35] Momayyezi M, Fallahzadeh H, Farzaneh F, Momayyezi M (2021). Sleep quality and cancer-related fatigue in patients with cancer. J Caring Sci.

[36] Mystakidou K, Parpa E, Tsilika E, Pathiaki M, Patiraki E, Galanos A (2007). Sleep quality in advanced cancer patients. J Psychosom Res.

[37] George GC, Iwuanyanwu EC, Anderson KO, Yusuf A, Zinner RG, Piha-Paul SA (2016). Sleep quality and its association with fatigue, symptom burden, and mood in patients with advanced cancer in a clinic for early-phase oncology clinical trials. Cancer.

[38] Shorofi SA, Nozari-Mirarkolaei F, Arbon P, Bagheri-Nesamie M (2021). Depression and sleep quality among Iranian women with breast cancer. Asian Pac J Cancer Prev.

[39] Al Maqbali M, Al Sinani M, Alsayed A, Gleason AM (2022). Prevalence of sleep disturbance in patients with cancer: a systematic review and meta-analysis. Clin Nurs Res.

[40] Sateia MJ (2014). International classification of sleep disorders-third edition: highlights and modifications. Chest.

[41] Lagergren P, Johar A, Liu Y, Ness-Jensen E, Schandl A (2022). Severe reflux and symptoms of anxiety and depression after esophageal cancer surgery. Cancer Nurs.

[42] Derakhshan MH, Malekzadeh R, Watabe H, Yazdanbod A, Fyfe V, Kazemi A (2008). Combination of gastric atrophy, reflux symptoms and histological subtype indicates two distinct aetiologies of gastric cardia cancer. Gut.

[43] Pengo MF, Won CH, Bourjeily G (2018). Sleep in women across the life span. Chest.

[44] Ayaki M, Tsubota K, Kawashima M, Kishimoto T, Mimura M, Negishi K (2018). Sleep disorders are a prevalent and serious comorbidity in dry eye. Invest Ophthalmol Vis Sci.

[45] Jongte L, Trivedi AK (2021). Alterations of the cardiovascular rhythms and sleep quality in esophageal cancer patients. Biol Rhythm Res.

[46] Chen D, Yin Z, Fang B (2018). Measurements and status of sleep quality in patients with cancers. Support Care Cancer.

[47] Morssinkhof MW, van Wylick DW, Priester-Vink S, van der Werf YD, den Heijer M, van den Heuvel OA (2020). Associations between sex hormones, sleep problems and depression: a systematic review. NeurosciBiobehav Rev.

[48] Kocevska D, Lysen TS, Dotinga A, Koopman-Verhoeff ME, Luijk M, Antypa N (2021). Sleep characteristics across the lifespan in 11 million people from the Netherlands, United Kingdom and United States: a systematic review and meta-analysis. Nat Hum Behav.

[49] Ratcliff CG, Zepeda SG, Hall MH, Tullos EA, Fowler S, Chaoul A (2021). Patient characteristics associated with sleep disturbance in breast cancer survivors. Support Care Cancer.

[50] Tian Y, Li LM (2017). [Epidemiological study of sleep disorder in the elderly]. Zhonghua Liu Xing Bing Xue Za Zhi.

[51] Dhruva A, Paul SM, Cooper BA, Lee K, West C, Aouizerat BE (2012). A longitudinal study of measures of objective and subjective sleep disturbance in patients with breast cancer before, during, and after radiation therapy. J Pain Symptom Manage.

[52] Savard J, Simard S, Blanchet J, Ivers H, Morin CM (2001). Prevalence, clinical characteristics, and risk factors for insomnia in the context of breast cancer. Sleep.

[53] Rissling MB, Liu L, Natarajan L, He F, Ancoli-Israel S (2011). Relationship of menopausal status and climacteric symptoms to sleep in women undergoing chemotherapy. Support Care Cancer.

[54] Tworoger SS, Davis S, Vitiello MV, Lentz MJ, McTiernan A (2005). Factors associated with objective (actigraphic) and subjective sleep quality in young adult women. J Psychosom Res.

[55] Wirth MD, Hébert JR, Hand GA, Youngstedt SD, Hurley TG, Shook RP (2015). Association between actigraphic sleep metrics and body composition. Ann Epidemiol.

[56] Endeshaw D, Biresaw H, Asefa T, Yesuf NN, Yohannes S (2022). Sleep quality and associated factors among adult cancer patients under treatment at oncology units in Amhara region, Ethiopia. Nat Sci Sleep.

[57] Hofmeister D, Schulte T, Mehnert-Theuerkauf A, Geue K, Zenger M, Esser P (2022). The association between sleep problems and general quality of life in cancer patients and in the general population. Front Psychol.

[58] Hsu MF, Lee KY, Lin TC, Liu WT, Ho SC (2021). Subjective sleep quality and association with depression syndrome, chronic diseases and health-related physical fitness in the middle-aged and elderly. BMC Public Health.

[59] von Ruesten A, Weikert C, Fietze I, Boeing H (2012). Association of sleep duration with chronic diseases in the European Prospective Investigation into Cancer and Nutrition (EPIC)-Potsdam study. PLoS One.

[60] Lee K, Cho M, Miaskowski C, Dodd M (2004). Impaired sleep and rhythms in persons with cancer. Sleep Med Rev.

[61] Berger AM (1998). Patterns of fatigue and activity and rest during adjuvant breast cancer chemotherapy. Oncol Nurs Forum.

[62] Ancoli-Israel S, Cole R, Alessi C, Chambers M, Moorcroft W, Pollak CP (2003). The role of actigraphy in the study of sleep and circadian rhythms. Sleep.

[63] Berger AM, Parker KP, Young-McCaughan S, Mallory GA, Barsevick AM, Beck SL (2005). Sleep wake disturbances in people with cancer and their caregivers: state of the science. Oncol Nurs Forum.

